# Periodic Tubular Structures and Phononic Crystals towards High-Q Liquid Ultrasonic Inline Sensors for Pipes

**DOI:** 10.3390/s21175982

**Published:** 2021-09-06

**Authors:** Nikolay Mukhin, Ralf Lucklum

**Affiliations:** Institute for Micro and Sensor Systems, Otto-von-Guericke-University Magdeburg, 39106 Magdeburg, Germany; ralf.lucklum@ovgu.de

**Keywords:** acoustic sensor, phononic crystal sensor, acoustic and elastic waves, quality (Q)-factor, acoustic liquid resonances in pipes, radial resonant mode

## Abstract

The article focuses on a high-resolution ultrasound sensor for real-time monitoring of liquid analytes in cylindrical pipes, tubes, or capillaries. The development of such a sensor faces the challenges of acoustic energy losses, including dissipation at liquid/solid interface and acoustic wave radiation along the pipe. Furthermore, we consider acoustic resonant mode coupling and mode conversion. We show how the concept of phononic crystals can be applied to solve these problems and achieve the maximum theoretically possible Q-factor for resonant ultrasonic sensors. We propose an approach for excitation and measurement of an isolated radial resonant mode with minimal internal losses. The acoustic energy is effectively localized in a narrow probing area due to the introduction of periodically arranged sectioned rings around the tube. We present a sensor design concept, which optimizes the coupling between the tubular resonator and external piezoelectric transducers. We introduce a 2D-phononic crystal in the probing region for this purpose. The Q-factor of the proposed structures show the high prospects for phononic crystal pipe sensors.

## 1. Introduction

Ultrasonic sensors are well-developed instruments and hold a long tradition. They are used for non-destructive, non-invasive and accurate measurements of technical and bio-liquids with a broad variety of chemical/biological compositions [1] as well as for many other applications with traditional and new transducer devices, just recently e.g., [2,3,4,5,6,7,8,9,10,11]. In contrast to acoustic microsensors such as the quartz crystal microbalance (QCM), they apply longitudinal acoustic waves which are able to propagate through the whole volume of matter. When impinging interfaces (large compared to wavelength), the waves are partly transmitted and reflected. When an acoustic wave propagates through a liquid sample, its amplitude decreases and the phase changes. The decline in the ultrasound amplitude or intensity is commonly termed attenuation. The phase is linked to the velocity of the propagation of sound through a sample. Physicochemical features of liquids govern variations of sound velocity and attenuation. By measuring the acoustic parameters, specific information about the properties of a liquid sample can be accessed. A previous paper already shows that the speed of sound can provide information about the composition of a liquid [12]. Meanwhile, measuring the speed of sound has become a standard method. Molecular interactions result in excess compressibility and excess volume [13]. Consequently, the speed of sound contains information about the nature of interactions between molecules, changes in the shape of molecules, or the type of interaction potentials between the components of a liquid mixture [14]. The speed of sound of various binary, ternary and multi-component liquid mixtures with completely soluble components has recently been reported in large number of works [15,16,17,18]. Furthermore, the speed of sound in suspensions and emulsions has been studied in detail in [19]. While speed of sound predominantly reflects changes in the chemical, ionic and molecular composition of the liquid, ultrasound attenuation is primarily sensitive to liquid bulk and shear viscosity. Moreover, attenuation contains information on thermal conductivity, density, and the specific heat of the liquid (see later in the text). Finally, attenuation responds to particles present in the liquid medium. It senses changes at the nanometer and micrometer size level and is suitable for characterizing liquid properties associated with inhomogeneity and phase composition of dispersed liquid systems, e.g., [20,21].

A typical method for measuring the bulk properties of liquids is using the resonances of acoustic waves in liquid-filled cavities. Resonators can be of rectangular or cylindrical shape, made of stiff solid materials (such as stainless steel or glass). The shift of the resonance frequencies of standing waves in a liquid-filled cavity is strongly related to a change in the speed of sound of the liquid (mixture). The loss factor (the reciprocal of the Q-factor) is associated with the attenuation of acoustic waves in the liquid volume. However, various types of other loss, specifically at the solid liquid interface, acoustic wave radiation into the liquid volume outside the resonator cavity, acoustic mode coupling and mode conversion must be taken into account. Without special measures, these mechanisms can become dominant, larger than the intrinsic liquid damping in the resonator chamber.

This paper focuses on a high-resolution ultrasonic sensor for real-time monitoring of liquid analytes in cylindrical pipelines. The cylindrical shape (pipelines, tubes, capillaries etc.) is the most natural shape when working with liquids. The development of sensors which are adapted to tubes is a current demand in medicine and biology (diagnostics of biological fluids in real time), in the food and chemical industries (online monitoring of the composition of liquid in pipes and microreactors at different stages of the process), or in mechanical engineering (analysis of fluids in fuel supply systems). As known, various acoustic resonance modes can be excited in a cylindrical resonator. Longitudinal, azimuthal, and radial modes are the most important ones [22]. Our study shows that the radial mode provides the best condition to achieve high values of the Q-factor of resonances, and, consequently, high sensitivity of the sensor to the bulk properties of liquids.

The development of a highly sensitive tubular sensor faces a number of questions:

- Excitation of the radial mode turns out to be the optimal one in comparison with other modes, hence, how can the radial mode in a long channel be excited and readout?;
- How can this mode be sufficiently isolated to avoid disturbances by other modes?;
- Acoustic (resonance) energy can be radiated along the pipe axis, hence, how can acoustic energy be localized in a small resonant liquid region within a pipeline?;
- The piezoelectric transducers such as PZT have usually a broad resonance, hence, how can the optimal coupling between the high-Q liquid resonator and piezoelectric elements be achieved?

In this work, we present a solution to the aforementioned problems by using the concept of phononic crystals. Phononic crystals (PnC)s are periodic composite materials with spatial modulation of elasticity, mass density, and longitudinal and transverse velocities of elastic waves. The typical structure consists of scattering centres with elastic properties which differ from the homogeneous matrix surrounding the scatters [23,24]. PnCs have been broadly used to control, direct, and manipulate sound waves. Their acoustic properties are defined by material parameters of the structure constituents, at least two materials with different acoustic properties, and by the structure design (geometry, symmetry, and periodicity) [25,26,27]. Variation of one material (or geometric) property of the PnC causes a change in the transmission behaviour of the whole structure. This opens several access points for phononic crystal sensors [28]. Nowadays, one typically designs a defect in an otherwise regular structure that causes isolated defect modes inside the band gap of the phononic crystals. The case of a phononic crystal with a liquid component is of special interest. We have introduced the commonly applied liquid phononic crystals sensor concept, featuring a defect. Meanwhile, several reports are available, ranging from proof-of-concept and new designs or concepts [29,30,31,32,33,34,35,36,37], to consideration of experimental influences [38,39] and 3D printing [40,41], to the measurement of real samples [42,43]. Typical frequencies range from several hundred kHz to a few MHz, the respective dimensions of the phononic crystal from units of millimetres to 100 µm. However, a surface acoustic wave phononic crystal mass sensor with nanometric periodic grating and operating frequency above 100 GHz has also been proposed [44]. In our previous phononic crystal liquid sensors work, we have demonstrated that the peak frequency related to the defect mode depends on the speed of sound of the liquid confined in the defect. The sensitivity of sound velocity measurements, based on phononic crystals, can compete with well-established ultrasonic principles.

Tubular phononic crystals are a fundamentally new sensor design concept for in-line monitoring of liquids in cylindrical structures such as vessels, pipes, tubes or capillaries. This type of phononic crystal is created by a radical change in lattice geometry from 2D planar or 3D Cartesian, with translational symmetry to 3D cylindrical with both translational and rotational symmetries. This novel concept exploits a new way of controlling acoustic fields in liquids and elastic fields in solids in order to realize the most optimal sensing field in the liquid. Acoustic waves are tailored so that they perturb sub-volumes of the liquid in the pipe, revealing their physical properties. We have introduced the idea of using tubular phononic crystals in [45] and further developed in [46,47]. The major disadvantage of the previously developed solutions has been the limited sensitivity of the longitudinal mode due to high shear viscosity losses at liquid/solid interface, resulting in a lower Q-factor of resonance. In this paper, we propose excitation and measurement of an isolated radial resonant mode with minimal internal losses in a liquid-filled pipe. It requires a new sensor design. We show how the concept of phononic crystals allows for an effective localization of the acoustic energy in a narrow probing area, thereby achieving the maximum theoretically possible Q-factor for ultrasonic measurements of liquids in tubes. We propose a combination of a 1D- and a 2D-PnC approach for excitation, detection and confinement of the radial mode and specifically introduce the periodically arranged sectioned rings around the tube as key element.

Another beneficial engineering consequence should be mentioned: the sensor measures across the pipe, not along the pipe. The design neither requires any mechanical obstacle inside the pipe nor transducer elements at one or both ends of the pipe. In terms of a (micro)fluidic system, all elements of the 1D- and the 2D-PnC, as well as the piezoelectric transducers, are mounted at the outer rim of the pipe; thus, i.e., the fluid can freely flow through the pipe.

## 2. Materials and Methods

### 2.1. Approach and Main Objectives

Resonant acoustic sensors achieve high sensitivity by optimal selection and successful excitation and detection of the resonant mode as well as its isolation from other vibration modes of the structure. The Q-factor of the sensor increases by minimizing all kinds of loss mechanisms. The realization of a high-Q tubular acoustic sound velocity sensor involves several challenges. Technical tubes are long and open systems. A liquid-filled tube has many different resonance modes; their characteristics are determined not only by the conditions of the propagation of acoustic waves in the solid and liquid parts, but also by liquid/solid interactions. Challenges arise from finding a way to excite the optimal high-Q mode and its isolation and effective readout, and also from the necessity to suppress acoustic loss mechanisms caused by radiation, mode conversion and coupling as well as losses at the solid/liquid interface.

In this study, we propose an approach that aims to improve the performance of a tubular resonant sensor using phononic crystal concepts. Periodically modulated acoustic properties in space result in real and virtual reflective boundaries for sound waves. They control the acoustic wave interference conditions and propagation of sound at specified frequencies. With this approach, the geometry becomes the key to tune the basic characteristics of the sensor system.

It implies the fulfillment of the following requirements:

- the type of resonant mode of liquid pressure in the channel should provide minimal shear viscous losses;
- the acoustic wave energy should be focused in a narrow section of the pipe channel, in which resonance measurements are carried out;
- losses due to the radiation of acoustic energy into the passive parts of the sensor and (micro)fluidic structure should be eliminated;
- acoustic measurements in flow systems should be realized without creating additional local hydraulic resistances.

A theoretical analysis of the sensor structure and its components was carried out on the basis of numerical simulation using COMSOL Multiphysics software.

### 2.2. Acoustic Pipe-Based Resonator Designs

The key element of the acoustic sensor is the liquid cavity resonator. In this article, we have explored tubular resonators and how they must be designed in order to reach the maximum Q-factor. At the same time, the inner cavity has a cylindrical shape, is filled with a liquid, has a considerable length with a constant inner diameter, is without any obstacles, and does not contain any technological holes or any other elements. This corresponds to the requirement that the investigated liquid can flow freely through the tube without encountering any additional hydraulic resistances.

**Figure 1:**
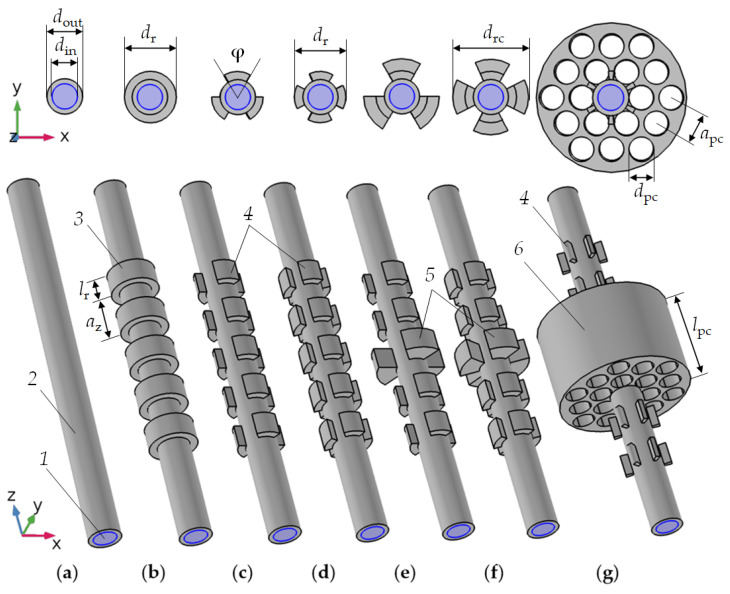
Model development of pipe-based resonators: a long liquid-filled pipe (**a**); a tubular phononic crystal with full (**b**) and sectioned rings periodically arranged along the pipe axis (3 (**c**) and 4 (**d**)); tubular phononic sectioned rings, where the central ring sectors have a larger diameter (**e**,**f**); a tubular phononic crystal acting as a defect in a two-dimensional phononic crystal (**g**). Resonator models consist of the following elements: *1*—liquid; *2*—pipe; *3*—identical rings; *4*—equidistant sectioned rings; *5*—sectioned rings of a larger size in the centre of the pipe; *6*—2D phononic crystal made of a perforated cylindrical block.

Figure 1 shows the pipe-based resonator structures in the order of their complexity (from left to right). In this order, the article will consider the approaches to increase the Q-factor of tubular resonators and, consequently, the resolution of the pipe-based acoustic sensor to a change in the speed of sound in the liquid analyte.

Figure 1 demonstrates the conceptual transition from a tubular resonator (Figure 1a) to a regular tubular phononic crystal (Figure 1b–d), a tubular phononic crystal with a defect in the middle along the pipe (Figure 1e,f), and finally to a combination of a 1D- and a 2D-phononic crystal on a pipe base (Figure 1g).

Figure 1a shows a tube filled with liquid (*1*). The pipe material (*2*) is coloured in gray. It can be glass or stainless steel or another solid material with low acoustic losses. Such a simple tube is characterized by two geometrical parameters: the inner and outer diameter (*d*_in_ and *d*_out_). The total length of the tube is much larger than its diameter. In Figure 1b, the tube has been extended by an arrangement of outer equidistant rings of diameter *d*_r_ and length *l*_r_, arranged in the direction of the tube axis to realize a 1D-phononic crystal with lattice period *a*_z_. The purpose of the 1D-PnC is to control the propagation of acoustic waves along the pipe. The number of rings *n*_r_ can be different. Figure 1b shows only 5 rings to prevent overloading the drawing. In reality, more rings may be required to provide optimal conditions for acoustic wave Bragg reflection [48]. By dividing the rings into *n*_s_ equal-equidistant sectors one gets the structures in Figure 1c,d, where *n*_s_ is equal to 3 and 4, respectively. Sectioned rings significantly expand the possibilities of tubular photonic crystal properties tuning. All sectioned rings can be of same size (Figure 1c,d), or the central part can be enlarged (Figure 1e,f). The latter provides easier access for excitation and readout elements. The last step of this development is the replacement of the central part of the 1D-PnC by a two-dimensional phononic crystal with a periodic arrangement of cylindrical empty holes (Figure 1g). This 2D-PnC is characterized by the type of symmetry (here hexagonal), the distance between adjacent holes (*a*_pc_) and their diameter (*d*_pc_). The design of a three-dimensional phononic crystal around the tube is not limited to the to the intention of Figure 1g; it offers potential to create various shapes and symmetries. The materials of the tube and the rings surrounding it can be different. They should have low acoustic losses, such as metals and glasses with a homogeneous microstructure. Our current sensor fabrication envisages prefabricated elements rather than a monolithic realization. The joints should have high quality. The assembly should avoid any glue, since it introduces a material with a significantly different (usually complex) acoustic impedance.

### 2.3. Physical Aspects

The acoustic pressure time fluctuation in liquid is governed by the wave equation [49]:

(1) $$\left(\frac{1}{v^{2}}\frac{\partial^{2}}{\partial t^{2}}-\nabla^{2}\right)p\left(r,t\right)=0,$$

where ∇ is the Nabla operator; *v* is the speed of sound in a liquid; *p* is the pressure; **r** = (*x*, *y*, *z*) is the coordinate vector; *t* is the time.

Liquid pressure resonances in pipes can be described based on the Helmholtz equation. Resonance modes can be found by solving the eigenmode problem for acoustic modes in a cylindrical cavity. The basic equation for the pressure wave with harmonic solutions (the Helmholtz equation) in the cylindrical coordinates is:

(2) $$\left[\frac{\partial^{2}}{\partial r^{2}}+\frac{\partial }{r\partial r}+\frac{\partial^{2}}{r^{2}\partial \theta^{2}}+\frac{\partial^{2}}{\partial z^{2}}+{\left(\frac{2\pi f}{v}\right)}^{2}\right]p\left(r,\theta ,z\right)=0,$$

where r,θ,z are radial distance, azimuth and distance from the *z*-axis; *f* is the frequency.

Applying the boundary conditions to Equation (2) for the cylindrical cavity of diameter *d* and length *L*:

(3) $$p\left(r,\theta ,z\right)=p\left(r,\theta +2\pi ,z\right);\;\left(\frac{\partial }{\partial r}+\frac{1}{r}\right)p\left(\frac{d}{2},\theta ,z\right)=0;\;\frac{\partial }{\partial z}p\left(r,\theta ,-L\right)=0,$$

the following solutions for the pressure distribution in a cylindrical resonator and the corresponding resonance frequencies can be obtained [50]:

(4) $$p_{l,m,n}\left(r,\theta ,z\right)=P_{l,m,n}J_{m}\left(\xi_{l,m}r\right)\cos\left(m\theta -\pi /2\right)\cos\left(n\pi z/L\right);\;f_{l,m,n}=\frac{v}{2\pi }\sqrt{\xi_{l,m}^{2}+{\left(\frac{n\pi }{L}\right)}^{2}},$$

where *l*, *m* and *n* are integers ≥ 0; *P* is the pressure magnitude, *J* is the Bessel function; the parameter values $\xi_{l,m}$ can be found from the numerical solution of Equation (5):

(5) $$J_{m}\left(\xi_{l,m}d/2\right)/J_{m+1}\left(\xi_{l,m}d/2\right)=\left(\xi_{l,m}d/2\right)/\left(m+1\right).$$

Parameters *l*, *m* and *n* in Equations (4) and (5) number radial, azimuthal and longitudinal eigenmodes.

In practise, the pressure distribution in liquids and the resonance eigenfrequencies in a cylindrical resonator differ from the analytical solution in Equation (4), since elastic waves in the solid body of the resonator were not considered there. To take into account a solid-liquid interaction, the Equation (1) must be self-consistently solved with the equation of propagation of acoustic waves through solid media:

(6) $$\rho \frac{\partial^{2}u_{i}\left(r,\;t\right)}{\partial t^{2}}=\sum_{j}\frac{\partial T_{ij}}{\partial x_{j}^{}},$$

where ρ is the density; *u_i_* are the components of the elastic displacement field; *T_ij_* are the stress tensor components; *i* and *j* are indices running from 1 to 3.

Conditions at the boundaries of the solid-liquid section are as follows:

(7) $$F=-n_{s}p;\;\left(n_{f}\cdot u\right)\omega^{2}=-n_{f}\left(-\frac{1}{\rho }\nabla p+q\right),$$

where **n**_s_ is the normal vector directed away from the solid part; **F** is the force per unit area representing the load on the cylinder walls; **n**_f_ is the normal vector directed away from the liquid volume; **u** is the mechanical displacement vector in the solid; **q** is the acceleration vector reported by the liquid.

Until now, we have not taken into account the problem of dissipation of the energy of acoustic waves. In order to simulate a real situation, we must introduce these losses into physical and mathematical models.

Mathematically, damping can be taken into account using an approach that involves a complex representation of time harmonic fields *A*(**r**, *t*):

(8) A(r,t) = Re[A(r)eiωt],

where *A*(**r**) is a complex-valued magnitude as a function of the position vector; ω = 2π*f*. Dissipation can be modelled by a complex speed of sound for liquids and a complex acoustic impedance for solids. The ratio between the total stored and the total lost energy in a device depends not only on the individual loss terms but also on the energy distribution within the device [51]. This explains why each resonance mode can have a different Q-factor. It can be calculated as a ratio of stored energy to energy lost per cycle, or as the resonance frequency of the peak divided by its full width at half maximum:

(9) $$Q\;=2\pi \frac{W_{\mathrm{st}}}{w_{\mathrm{loss}}^{c}};Q\;=\frac{f_{\mathrm{res}}}{\Delta f_{\mathrm{FWHW}}},$$

where *W*_st_ is the energy stored in the system; $w_{\mathrm{loss}}^{c}$ is energy loss per cycle; *f*_res_ is the resonance frequency; $\Delta f_{\mathrm{FWHW}}$ is the full width at half maximum of the resonance peak. Considering different loss mechanisms:

(10) $$\frac{1}{Q}=\sum_{j}\frac{1}{Q_{j}},$$

hold, where *Q_j_* is the *j*^th^ loss mechanism.

The damping mechanisms important for acoustic tubular liquid sensor structures are (a detailed description of the damping mechanisms can be found in the review [51]):

- Bulk losses in liquids due to viscosity. The bulk viscosity plays an important role for ultrasonic wave propagation in most liquids and is associated with damping of longitudinal waves. The dynamic viscosity also contributes to volumetric losses;
- Thermal damping in the bulk of liquid. Any acoustic field induces a fluctuating temperature field due to thermoacoustic coupling. In the bulk, this fluctuating temperature field is proportional to the acoustic pressure. Thermal conduction in the liquid bulk leads to a loss factor, which causes the so-called thermoelastic damping. It depends on the ratio of specific heats, the heat conductivity and the specific heat at constant pressure;
- Viscosity loss at the walls of resonator cavity, i.e., at liquid/solid interfaces. A viscous boundary layer on the walls of a liquid-filled cavity is the main source of dissipation. Due to the no-slip boundary condition at the interface between the solid and the liquid, the viscous boundary layer is a transition layer in which the tangential component of the liquid velocity adapts to the velocity of the solid boundary. During each cycle, the viscous shear in this layer dissipates a fraction of the acoustic energy. In terms of the sensor, the amount depends on both the resonance mode and the shape of the cavity;
- Thermal damping at the liquid/solid interface. A thermal boundary interaction creates a thermal boundary layer and contributes to dissipation of acoustic resonance energy;
- Radiation losses. A pipe that the sensor is intended for has a much larger length than the length of the sensor. Attempts to excite acoustic resonance in some part of the pipe inevitably leads to the fact that part of the resonance energy will spread out along the axis of the pipe. Part of the ultrasonic energy will radiate into passive volumes of the system and into the environment;
- Losses due to particles suspended in the liquid. They depend on particle size (compared to the ultrasonic wavelength) and their mechanical properties. Consideration should be given to scattering and thermo-viscous dissipation at the particle-liquid interface. We do not consider damping due to suspended particles, since we are studying homogeneous liquids. Nevertheless, a good Q-factor of the tubular acoustic sensor extends the possibilities of characterizing the presence of particles in the analyte by the width of the resonance peaks and to estimate their concentration, particle size distribution, etc., see e.g., [52];
- Losses in solid materials. They depend on the constitutive behaviour of the solid material; viscoelasticity and thermoelasticity are the sources of acoustic wave energy dissipation. Furthermore, piezoelectric elements introduce dielectric and piezoelectric dissipation, leading to a complex dependence on the conditions of motion and vibration mode. Material behaviour, including damping, can vary significantly with temperature and frequency. In addition to volume scattering, energy is also lost from the surfaces of the device.

Using materials such as stainless steel or glass to build the resonator, losses in a solid part of a system can be safely neglected since the viscosity of these materials is extremely low. It should be noted that solid materials must satisfy the condition of homogeneity, where internal structural inhomogeneities are much smaller than the acoustic wavelength. From this point of view, 3D printing technologies may currently not be useful.

In addition, several aspects should be noted:

- Scaling. One of the advantages of resonant (sensor) principles is the tight correlation between resonance frequency and resonator dimension. Therefore, many (theoretical) papers apply reduced values, e.g., a normalized frequency scale. However, this concept fails if frequency-dependent effects such as relaxation phenomena (volumetric) or size-to-wavelength dependent effects, such as stick-slip or wetting phenomena (interfacial) or surface roughness (fabrication) issues, become significant;
- Excitation and readout electronics. Our high-Q resonant sensor requires a circuitry able to measure the resonance frequency and the respective half-bandwidth with high accuracy. During development and in lab environment a (commercial) impedance analyser is an appropriate solution. We have developed miniaturized solutions specifically designed for resonant sensors [53], both network analysis- [54] and oscillator-based [55]. The impedance mismatch between circuitry and electromechanical transducer [56] is influenced by the acoustic impedance mismatch of transducer, PnC and pipe materials and the liquid and their respective effective acoustic impedances. We concentrate on the acoustic path here.

When comparing all of the possible sources of acoustic energy loss, viscosity of the liquid and radiation of acoustic energy along the pipe are the most important loss mechanisms and the most important challenge to enhance the sensor’s resolution.

To consider the losses in the liquid bulk, a general description of the propagation of sound in a compressible fluid can be conducted using the Navier-Stokes and continuity equations [57]. In addition, the equations of energy balance and thermodynamics [58] must be used, which describe the energy of the system based on state variables, i.e., temperature, pressure, and density. Considering only terms of the first order in time and harmonic solutions, the following equations can be obtained [58]:

(11) $$\omega^{2}v+\left[\frac{v^{2}}{\gamma }-i\omega \frac{\eta }{\rho }\left(\frac{3}{4}+\frac{\eta_{B}}{\eta }\right)\right]\nabla \left(\nabla \cdot v\right)+i\omega \frac{\eta }{\rho }\nabla \times \nabla \times v=\frac{i\omega \beta v^{2}}{\gamma }\nabla T;$$

(12) $$\gamma \frac{\kappa_{\mathrm{th}}}{\rho C_{p}}\nabla^{2}T+i\omega T=\frac{\left(\gamma -1\right)}{\beta }\nabla \cdot v,$$

where **v** is the velocity vector of the liquid; *T* is the temperature; γ is the coefficient of specific heat, κth is the thermal conductivity; ρ is the density; *C*_p_ is the specific heat at constant pressure; β is the volumetric compressibility; η is the shear viscosity; η_B_ is the bulk viscosity; *v* is the speed of sound. The volumetric and shear viscosity as the dissipation coefficients for the compressive motion of the fluid are associated with friction. Bulk liquid losses are the most fundamental natural limitation of the sensitivity of acoustic liquid sensors.

Viscosity and thermal damping at the liquid/solid interface can be considered by the self-consistent solution of the Equations (6), (11) and (12). These losses occur in the near-surface layers of the liquid of the following depths:

(13) $$\delta =\sqrt{\frac{\eta }{\pi \rho f}};\;\delta_{\mathrm{th}}=\sqrt{\frac{\kappa_{\mathrm{th}}}{\rho C_{p}f}},$$

where δ and δ_th_ are the viscous and thermal penetration depths, respectively.

To minimize damping at the liquid/solid interface, we have searched for the most optimal mode in a tubular resonator, i.e., one which does not show shear displacement at this interface.

To minimize radiation losses, along with losses at interfaces, we propose a sensor resonator structure based on Figure 1d. This phononic crystal structure should prevent radiation losses, i.e., it should realize two virtual boundaries along the pipe, which localize the selected resonance mode in a certain part of the pipe along its length.

To sum up, we cannot neglect acoustic energy losses in the liquid and at the solid/liquid interface. They are significant and determine the Q-factor of the resonance. However, besides the sound velocity of the liquid, liquid viscosity provides additional independent and hence important information about the liquid in the pipe. Still, liquid bulk viscosity contains already this information, hence shear viscosity at the solid/liquid interface is redundant and should be avoided, Task 1. We propose the radial mode realizing a sharp sensor resonance.

Acoustic energy radiation should be avoided by an appropriate 1D-phononic crystal design along the pipe, Task 2. We propose the structure shown in Figure 1g. Furthermore, it should optimize the coupling between the tubular resonator and the piezoelectric transducer.

## 3. Results

### 3.1. Liquid-Filled Cylindrical Resonator

Since the Q-factor is the most important parameter for the resolution of the resonant sensor, the following questions are of great interest: how does the Q-factor of liquid resonances in a pipe depend on the type of acoustic eigenmode, frequency and pipe diameter? How can the corresponding resonant modes be excited and what is their sensitivity to changes in the speed of sound? How do solid/liquid interfaces affect the Q-factor of resonances?

The infinitely long liquid-filled cylindrical resonator, as shown in Figure 1a, was considered as a starting point for the study. At this step, we assume that there are no losses due to radiation of acoustic energy. The mechanical effect that excites resonant modes acts along the entire length of the tube. Furthermore, acoustic losses of the solid pipe material are negligible. Thus, all of the effects that are obtained in this paragraph by simulation are associated with losses within the liquid bulk and at the solid liquid interface.

**Figure 2:**
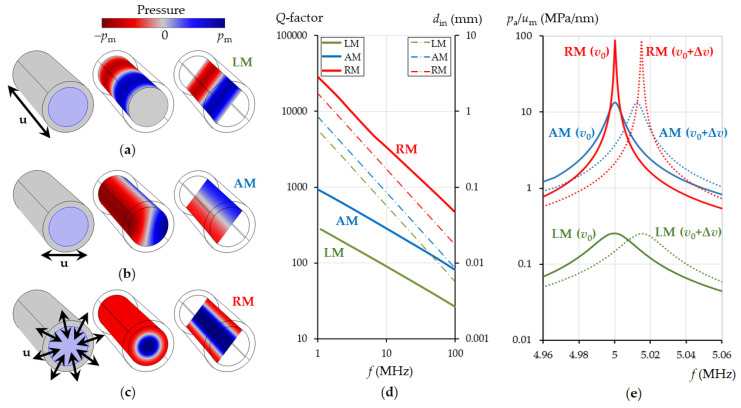
Resonant mode excitation in a cylindrical resonator and their Q-factors: pressure distribution in a liquid for the longitudinal (**a**), azimuthal (**b**) and radial (**c**) mode according to the action of mechanical displacement in the solid as shown by arrows. Dependence of the Q-factor of the eigenmodes on the resonance frequency (**d** left axis) and the corresponding values of the inner diameter of the cylindrical resonator (**d** right axis). Frequency dependence of the volume-averaged absolute pressure in the liquid in relation to the magnitude of the exciting mechanical displacement at 5 MHz resonance (**e**). The calculations were performed for water at a temperature of 20 °C, *v*_0_ = 1481 m/s (solid lines), Δ*v* = 5 m/s (dotted lines).

By means of external mechanical action in such a resonator, it is possible to excite various acoustic modes. The three main, fundamental eigenmodes of a cylindrical resonator were selected: the longitudinal (LM), azimuthal (AM) and radial (RM) liquid pressure modes. All of the other modes are either higher harmonics or a superposition of these fundamental modes. Therefore, by understanding the behaviour of these basic modes, we can extrapolate this knowledge to other cases. We are specifically interested in what are the theoretical limits of the Q-factor of these resonant modes and how the Q-factor changes depending on frequency. The temperature was taken equally at 20 °C. Water was taken as a liquid (parameters are summarized in Table 1). A solid substance with mechanical properties close to silicon was taken as the material of the cylinder walls: Young’s modulus is 163 GPa, Poisson’s ratio is 0.22, density is 2330 kg·m^−3^. The numerical calculation was carried out by the finite element method based on the self-consistent solution of the Navier-Stokes equations for a liquid and the equation of propagation of elastic waves in a solid. The mesh size for the finite element method was considered as the viscous and thermal damping at the liquid-solid interface, which is significantly less than the penetration depth of shear waves into the liquid (see Equation (13)). To excite a longitudinal mode (*l* = 0, *m* = 0 in Equation (4)), a vector of displacement (**u**) was applied to the liquid-filled cylindrical pipe parallel to its axis (Figure 2a). The magnitude of displacement (*u*_m_) was kept constant throughout the infinitely long pipe and acted in harmonic law at resonance frequency. To excite the first azimuthal mode (*l* = 0, *m* = 1, *n* = 0 in Equation (4)), the mechanical displacement vector was applied perpendicular to the pipe (Figure 2b). To excite the first radial mode (*l* = 1, *m* = 0, *n* = 0), the mechanical displacement vector was applied normal to the pipe surface (Figure 2c). The eigenfrequencies of the pipe resonator were calculated depending on its inner diameter. We took the thermal and viscous mechanisms of damping in the liquid bulk and at the solid liquid interface into account.

We found that the values of the Q-factor (Equation (9)) of the modes depend on their resonance frequency. The dependence of the Q-factor of each of the main eigenmodes on the frequency are shown in Figure 2d (thick solid lines). The red, blue and green colours of the lines correspond to the radial, azimuthal and longitudinal modes, respectively. The Q-factor drops dramatically with an increase in frequency. This dependence is linearized on a double logarithmic scale. The resonant frequency of the eigenmodes inversely depends on the inner diameter of the cylinder; therefore, the decrease in the Q-factor can be attributed to an increase in the influence of surface effects, friction at the interface between liquid and solid, as the pipe diameter decreases with increasing frequency. For convenience, the relationship between the inner diameter and the resonant frequency for each mode is shown in Figure 2d with thin dashed lines.

Figure 2e shows the resonance peaks of the three main modes and their frequency shift when the speed of sound in the liquid changes by 5 m/s. The results show that the radial mode is the most sensitive. The Q-factor is the highest, hence it allows the highest resolution to changes in the speed of sound in a liquid. The calculation was made for a frequency of 5 MHz (in Figure 2e the inner pipe diameter was selected for each mode so that its resonant frequency was 5 MHz at a sound speed of 1481 m/s in water), but the conclusion that the radial mode is the preferable mode for sensing is valid at all frequencies. The high-Q of the radial mode can be explained by the special vibrational regime of the solid and the pressure distribution in the liquid at which surface friction is negligible and surface losses are significantly reduced in comparison with the other cylindrical resonator eigenmodes.

### 3.2. Tubes with Periodically Arranged Rings. Tubular Phononic Crystals

In Section 3.1 it was shown that the radial mode has the highest Q and it should be chosen to realize a tubular acoustic sensor. However, we have considered the excitation of all modes along the entire length of the tube; therefore, radiation of acoustic energy along the axis of the tube was not taken into account. In reality, the region of excitation of resonance in the tube is limited by the geometry of the piezoelectric transducers, i.e., they have a finite (lateral) dimension. Since the propagation of acoustic waves along the pipe is not limited by anything, acoustic waves will be radiated out of the source area, which leads to a significant decrease in the resonance Q-factor. Therefore, this radiation loss devalues the benefits of the radial mode. How can this be prevented?

We propose to use the periodic arrangement of rings to create virtual boundaries realizing the Bragg-type reflection of acoustic waves propagating along the pipe. We apply the ability of phononic crystals to control and tailor elastic and acoustic wave propagation.

Given that the tubular phononic crystal is a periodic structure in z-direction, the Bloch theorem can be used to determine the eigen solutions. According to this theorem, the displacement vector can be represented as a product of a propagating wave and a periodic function describing the phononic crystal:

(14) $$u\left(r,k\right)=u_{k}\left(r\right)\exp\left(-ikr\right),u_{k}\left(x,y,z\right)=u_{k}\left(x,y,z+a_{z}\right),$$

where $u_{k}$ is a periodic function of **r**; **k** is the wave vector.

As previous results show [46], a phononic tubular crystal made of periodically spaced rings (Figure 1b) can effectively reflect acoustic waves and prevent their propagation along the tube. However, it works in a narrow frequency band only and it is inappropriate for dealing with the radial mode. For this reason, we refrained from full rings and went to the sectioned rings design (Figure 1c,d) of the tubular phononic crystal. The application of ring segments significantly expands the possibilities of controlling the propagation of acoustic waves in a pipe. It is better adapted to the radial mode because the configuration of the sectors corresponds to the natural way of exciting the radial mode by applying a normal mechanical displacement to each sector.

**Figure 3:**
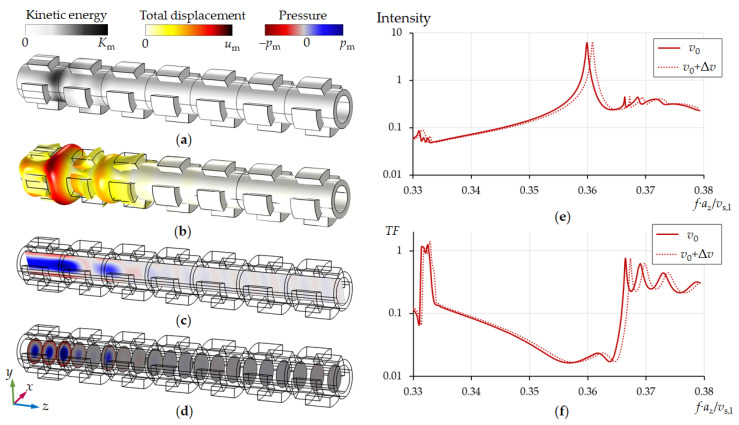
Propagation of acoustic waves along a pipe with a periodic structure of sectioned rings from left to right (**a**–**d**), the intensity of acoustic waves in the first unit cell (**e**) and the transmission spectra for two sound velocities of water (**f**). The inner diameter of pipe is *d*_in_ = 353 μm. Lattice period of a tubular phononic crystal is *a*_z_ = 2*d*_in_. The calculations were performed for *v*_0_ = 1481 m/s, Δ*v* = 5 m/s.

Figure 3 shows the propagation of acoustic waves through a pipe with a periodic layout of sectioned rings. The radial mode was excited by applying a mechanical displacement to each ring sector along the normal of the cylinder surface only in the first unit cell on the left side of the tube. The results show that it is possible to choose the parameters of the tubular phononic crystal in a way that the penetration depth of the acoustic wave along the tube axis is limited to the first two lattice periods (Figure 3).

In Figure 3e,f the frequency scale is normalized by multiplying by the lattice constant and dividing by the longitudinal speed of sound in solid ($v_{s,l}$), which is associated with the mechanical properties of a solid in the Equation (6):

(15) $$v_{s,l}={\left\{\left[E\left(1-\upsilon \right)\right]/\left[\left(1+\upsilon \right)\left(1-2\upsilon \right)\rho \right]\right\}}^{1/2},$$

where *E* is the Young’s modulus; υ is the Poisson’s ratio; ρ is density. We have chosen again the values of silicon, see above.

**Figure 4:**
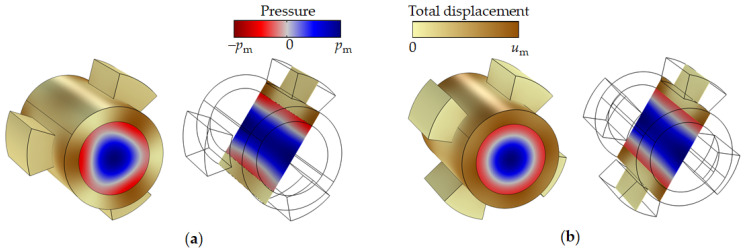
Unit cells of the tubular phononic crystal with a 3 (**a**) and 4 (**b**) ring segments: distribution of pressure and displacement fields. The radial mode was excited by applying a mechanical displacement to each sector along the normal of the cylinder surface.

Thus, the periodic arrangement of ring segments makes it possible to prevent radiation of acoustic energy along the pipe in a fairly wide frequency range, which can be tuned by the geometric parameters of the tubular phononic crystal (*d*_in_, *d*_out_, *d*_r_, *l*_r_, *a*_z_, *n*_s_, φ). The number of ring sectors (*n*_s_) can theoretically vary from 3 to infinity. An increase in *n*_s_ improves the pressure distribution in the pipe towards an ideal displacement distribution of the radial mode (see Figure 2c). However, for engineering reasons we have restricted *n*_s_ to 3 and 4. The latter already provides very reasonable results (Figure 4b). The Q-factor of the mode in Figure 4a is lower than in Figure 4b, since the radial mode in the first case is more distorted and, consequently, is subject of large losses due to friction at the liquid/solid interface. Calculations show that the resonance Q-factor increases with an increase in *n*_s_, however, with *n*_s_ > 4 these changes become insignificant.

### 3.3. Tubular Phononic Crystal Liquid Sensor

To create an effective tubular liquid sensor, it is necessary to excite and readout a high-Q well-isolated resonance mode at the centre of the tube. A standard way of realizing phononic crystal sensors is to create a defect in a periodic structure, the resonance frequency of which is within the bandgap of a regular phononic crystal, and to transmit acoustic waves through this structure [28]. Based on this principle, we have proposed the first tubular phononic crystal sensors [45]. However, following this approach, it is hardly possible to excite a high-Q radial mode. Therefore, we propose a different, more effective approach. It realizes the excitation and readout of the radial resonant mode by applying a force directly to the central unit cell of a tubular phononic crystal, following the results in Figure 4.

**Figure 5:**
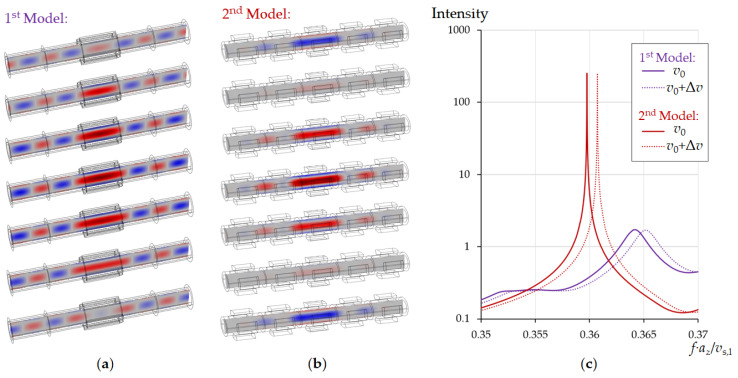
Snapshots of the pressure distribution in the pipe during one period of oscillation when excited in the center for the cases of a simple pipe (**a**) and a pipe surrounded by a periodic structure, (**b**) as well as comparison of the Q-factor of their resonances (**c**). The calculations were performed for water at a temperature of 20 °C, *v*_0_ = 1481 m/s, Δ*v* = 5 m/s. The inner diameter of pipe was *d*_in_ = 353 μm.

Since the central unit cell faces a phononic crystal both left and right, the acoustic energy of resonance will be localized within it. Radiation of acoustic energy along the pipe axis can be excluded and the resonance provides a high Q-factor. For illustration, we compare the two models in Figure 5. Model 1 (Figure 5a) shows a section of the cylindrical tube with four equidistant piezoelectric elements along the circumference of the pipe placed in the central part excites the radial resonance mode of liquid pressure. Model 2 (Figure 5b) considers a tubular phononic crystal of 5 sectioned rings. Diameter and length of the tubes are the same in both models. At the edges of the tube, the boundary conditions for the radiation of acoustic wave energy are applied. The central segments in both models are excited by applying an external mechanical displacement normal to the surface of the tube. Full harmonic oscillations of the liquid pressure in the pipe were calculated. Figure 5a,b show a series of images representing one oscillation period, divided into 7 snapshots. The pressure in the central part of the pipe was calculated (Figure 5c) for two different sound velocities of the liquid. The other liquid parameters were the same (Table 1). In the first case, a traveling pressure wave is generated in the tube along the axis in directions from the centre to the edges (Figure 5a), which causes a low resonance Q-factor due to radiation losses along the axis of the tube (Figure 5c, blue curves). In the case of model 2, the phononic crystal elements applied to the outer part of the tube lead to the formation of a standing wave along the tube axis. The acoustic resonance energy is localized in the central part of the tube, which makes it possible to achieve an extremely high Q-factor (Figure 5c, red curves). The Q is limited only by internal viscous losses in the liquid. The radial mode also minimizes these losses due to the absence of shear displacement at the interface to the pipe. The Q therefore provides a well-defined access to important liquid properties, Equations (11) and (12) in addition to sound velocity.

Thus, the use of phononic crystals placed on the outer surface of the pipe the liquid to be analysed is flowing through improves the Q-factor of the acoustic resonances by at least an order of magnitude. This sensor design allows measurements of liquid properties without creating any additional local hydraulic resistances.

### 3.4. Signal Excitation and Readout Method

The radial mode has a number of advantages. A remaining drawback is associated with the fact that this mode is more difficult to excite. Indeed, if we look at Figure 2a–c, we note that the azimuthal and longitudinal resonance modes are easily excited by applying mechanical displacement along or across the tube. By contrast, excitation of the radial mode requires mechanical displacement applied to the circumference of the pipe, i.e., along the normal of the cylinder. Forces must be equidistantly distributed, i.e., the forces must have axial symmetry.

**Figure 6:**
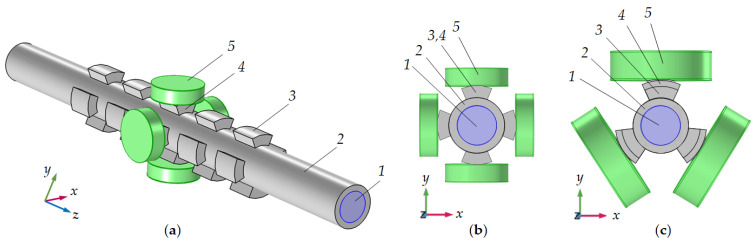
Radial mode excitation method: side (**a**) and front (**b**,**c**) views for the four (**a**,**b**) and three (**c**) sectioned rings tubular phononic crystals. *1*—liquid; *2*—pipe; *3* and *4*—regular and central sectioned rings; *5*—piezoceramic transducers.

By exploiting a tubular phononic crystal with sectioned rings, excitation of the centre unit cell can be carried out by direct contact piezoelectric transducers, attached to each ring sector. These identical piezoelectric transducers must operate synchronously in the extensional mode at the resonance frequency of a cylindrical liquid-filled cavity. Improved resonance is obtained using four or more segments, however, for a simpler mutual arrangement of piezoelectric elements in a limited space, their number can be reduced to three. For more convenient access to the central cell of a tubular phononic crystal, the segments in the center can be increased in the diameter. Figure 6 shows possible variants of the mutual arrangement of piezoceramic transducers. If the excitation of the radial mode is carried out by the synchronous operation of all piezoelectric elements, one of them is sufficient to measure the resonance frequency and to monitor its frequency and Q-factor shifts when the composition of the liquid changes. At this stage, we neglect non-ideal behavior of real piezoelectric transducers, e.g., caused by the finite lateral dimensions. Current research concentrates on multielectrode designs and high-Q piezoelectric materials.

### 3.5. Phononic Crystal Sensor

The major drawback of last layout is the limited control of the coupling between the ring sectors and the piezoelectric transducer, specifically matching of the impedances of the tube and piezoelectric elements. Another disadvantage of direct contact results from internal losses in piezoelectric elements, which are, to some extent, coupled into the high-Q liquid resonator. How can this be improved?

**Figure 7:**
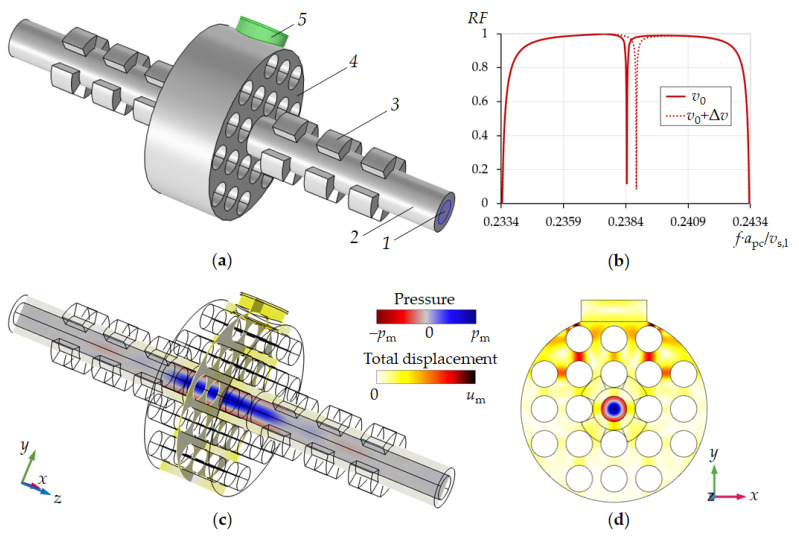
Design of the phononic crystal sensor, simulation results at resonance conditions in the pipe (**a**) with *1*—liquid, *2*—pipe, *3*—sectioned rings, *4*—2D phononic crystal, *5*—piezoelectric transducer. Reflection spectra of sensor (**b**). Pressure and displacement field distributions close to liquid resonance conditions (**c**,**d**).

We propose a combination of the 1D tubular phononic crystal with a two-dimensional phononic crystal (Figure 7). In this case, the tube resonator is surrounded by 1D-PnC reflectors along the pipe (direction of the tube axis) and a 2D-PnC normal to the pipe (transverse (or radial) direction). This design provides the best conditions for the concentration of acoustic resonance energy inside the sensor section of the tube and acoustic coupling with the piezoelectric elements.

The combination of a tubular periodic structure and a 2D-phononic crystal opens the gate to a liquid in-line measurement sensor with an extremely high resolution of liquid sound velocity as well as liquid viscosity. This result is achieved by reducing the energy losses of acoustic resonance due to viscosity of the liquid via the exploitation of the radial mode. Furthermore, the two tubular periodic structures suppress radiation of acoustic energy along the pipe while the 2D-phononic crystal realizes an optimal excitation and detection of the radial eigenmode of liquid pressure in a pipe by mode conversion and optimal coupling to the piezoelectric transducer. In addition, the 2D-phononic crystal allows the reduction in the number of piezoelectric elements to one.

Figure 7a shows the design of the new phononic crystal sensor. It consists of a liquid analyte-filled pipe surrounded by two phononic crystals: the one-dimensional tubular phononic crystal along the pipe and a two-dimensional cylindrical phononic crystal in the centre. Both the 1D- and the 2D-PnCs act mutually as defect in the particular other phononic crystal. One piezoelectric transducer has been mounted on the surface of the 2D-phononic crystal. The transducer vibrates in its extensional mode and acts both as a transmitter and as a receiver of acoustic waves. The 2D-phononic crystal has been designed in a way that it comprises a bandgap around the resonance of the piezoelement, i.e., the generated acoustic wave is reflected from the 2D-PnC at all frequencies within the bandgap, except the resonant frequency of the 1D-phononic crystal defect. This defect resonant frequency is that of the radial mode of a liquid-filled cylindrical cavity. The piezoelectric transducer therefore displays a sharp dip in its frequency response measured as the reflection coefficient of the piezoelectric transducer *RF* (Figure 7b). The resonant frequency of the radial mode of the liquid-filled cavity, i.e., the frequency of the dip in *RF* depends linearly on speed of sound of the liquid. Its bandwidth is a function of other liquid properties, first of all its viscosity (Equations (11) and (12)). Simulations show that the Q-factor of a given dip (Figure 7b) can be larger than the Q-factor of the resonance peak of the more simple tubular phononic crystal such as the one in Figure 5c. In fact, on the basis of this phononic crystal design, the limit of the acoustic resonant sensor’s sensitivity to the speed of sound in liquids can be reached.

## 4. Discussion

In a liquid-filled pipe, it is possible to excite various resonant modes by applying piezoelectric transducers to its wall. Longitudinal, radial and azimuthal resonance modes are sensitive to the speed of sound in a liquid. Consequently, it is possible to calculate the value of the speed of sound in a liquid analyte from the measured resonance frequency as well as parameters responsible for acoustic energy dissipation in the liquid from the bandwidth, first of all liquid viscosity. We have shown already that these mechanical values can be used for the determination of engineering or process data. However, the real cavity resonance frequencies differ from the ideal ones, even when neglecting dissipation. The cavity walls do not completely reflect the acoustic waves (otherwise, the sensor could not measure liquid properties). In terms of structural mechanics [59], the structural acceleration in the solid affects the liquid domain as an acceleration across the solid/liquid boundary. On the other hand, the liquid in contact with the vibrating solid structure exerts force in response to that vibration. This reaction is inertial in nature and physically associated with the kinetic energy of liquid motion in the proximity of the interface. One may draw the picture of a bi-medium oscillator, determined by two interrelated mechanisms: the inertial reaction of the liquid on the solid’s vibration and the elastic reaction of the solid on periodic pressure alternation on the solid-liquid interface. A pressure increase in the pipe must be balanced by a corresponding pressure decrease and finally a longitudinal stress in the adjoining solid volume to maintain equilibrium at resonance. The fact, that the solid is actually a phononic crystal, makes the analysis even more involved, since material properties must be replaced by effective acoustic properties.

As simulations show, the radial mode has the highest resolution of the speed of sound measurement. In addition to a slightly higher direct dependence on sound velocity, the Q-factor of the radial mode is by orders of magnitude higher, hence the Figure of Merit, FoM

(16) $$\mathrm{FoM}\;=\frac{\Delta f}{\Delta v\cdot \Delta f_{\mathrm{FWHW}}},$$

Is higher by orders of magnitude as well. The differences in the characteristics between the modes are determined by the acoustic losses at the solid/liquid interface of the tubular resonator. Our analyses have shown that shear viscous losses, associated with friction at the solid/liquid interface, are the most significant ones. For the radial resonance mode, the pressure distribution in the liquid is such that the maximum pressure is equidistant from its interface to the pipe and is close to the axis of the cylindrical pipe. Surfaces of equal phase are equidistant from solid/liquid interfaces, too. Therefore, the pressure gradient tangential to the solid wall is zero and shear viscous losses thus vanish. This makes the radial mode the most preferable for sensor applications.

Besides a (theoretically) high-Q pipe resonator, acoustic energy radiation out of the resonator volume along the pipe axis must also be avoided. In this study, we propose the application of a periodic arrangement of sectioned rings for realizing a Bragg-type reflection of acoustic waves in the liquid and in the solid propagating along the pipe axis. The key point is the fact that real reflective boundaries of the liquid inside the pipe must be avoided not to disturb a free flow of the liquid. All of the required structural elements are therefore located on the outer rim of the pipe. The inner surface of the pipe keeps absolutely smooth and even.

A tubular phononic crystal based on ring segments has proven its adaptability not only regarding the tuning of an acoustic bandgap, but it also turned out to be convenient in realizing the radial mode excitation and detection in a specific unit cell. A striking result is the fact that two periods to the left and right of the central cell are already sufficient in significantly improving the resonance characteristics. This corresponds to Figure 3, according to which the penetration depth of an acoustic wave into a phononic crystal can be less than two unit cells.

In contrast to the traditional approach in phononic crystal sensor literature, we propose a different, more effective approach, which consists in the excitation and readout of the radial resonant mode by directly addressing the acoustic field to the central unit cell of a tubular phononic crystal. The current state of this approach surrounds the center of a 1D-tubular phononic crystal by a 2D-phononic crystal. Furthermore, this approach allows optimization of the coupling of only one piezoelectric transducer with the acoustic field in the liquid-filled pipe resonator. This approach minimizes all the considered loss mechanisms and achieves an extremely high Q-factor.

## 5. Conclusions

The article critically analyses the idea and the physical principles of a novel acoustic liquid sensor concept. We present arguments supporting the proposed approach that combines ultrasonic measurement, resonant sensor and phononic crystal capabilities. Our alternative design of a tubular resonant acoustofluidic sensor uniquely combines the known properties of 1D- and 2D-phononic crystals for the benefit of concentrated acoustic energy in the measurement space.

As consequence of our analyses of the dominant mechanisms of losses in tubular liquid-filled resonators, the influence of acoustic mode regimes, solid-liquid interactions, geometric aspects and frequency dependencies, we have been able to propose an approach for excitation and measurement of an isolated radial resonant mode with minimal internal losses in a liquid-filled pipe. We could effectively localize the acoustic energy in a narrow probing area by the introduction of periodically arranged ring segments around the tube. The additional 2D-phononic surrounding probing region optimizes the coupling between the tubular resonator and an external piezoelectric transducer. Only one transducer is required, which should minimize the design of an electrical oscillator. The estimates of the theoretical Q-factor of the proposed sensor shows the high prospects for the pipe-based phononic crystal sensor.

The results of numerical studies show the following:

- The radial resonance mode, the pressure distribution in the liquid, the maximum pressure equidistant from the solid/liquid interface and close to the axis of the cylindrical pipe avoids shear viscous losses, usually most significant in acoustofluidic devices and associated with the friction at the liquid/solid interface. Surfaces of equal phase are equidistant from liquid/solid interfaces too. Therefore, the pressure gradient, tangential to the solid wall, is zero and shear viscous losses vanish;
- Our work extends the field of phononic crystal liquid sensors by a novel combination of 1D and 2D designs;
- A 2D-phononic crystal realizes the coupling with a single piezoelectric transducer;
- A 1D-phononic crystal composed of ring segments is suited to focusing on an isolated radial mode in a cylindrical tube, preventing radiation losses along the tube axis and providing access to a Q-factor of the acoustic resonances improved by orders of magnitude. The PnC requires elements on the outer surface of a cylindrical pipe only and does not introduce additional local hydraulic resistances;
- The sensor has a high potential in the field of liquid properties evaluation in microfluidic channels or macroscopic tubes or pipes e.g., in chemical industry.

Future research concentrates on two aspects of the error analysis: firstly, deviations from the ideal geometry, including surface roughness as well as diameter and layer thickness deviations along the pipe, and secondly, the influence of non-acoustic parameters, specifically temperature and pressure [46]. The activities include measurement system solutions with internal reference systems. Finally, the improvement of experimental studies is in permanent progress to achieve the required accuracy sufficient to validate the extraordinary sensitivity of the proposed sensor concept, i.e., a relative error specifically in geometry comparable to 1/Q.

## Figures and Tables

**Table 1:** Parameters of water at a temperature of 20 °C used in this study.

Parameters	Speed of Sound	Density	Shear Viscosity	Thermal Conductivity	Isobaric Heat Capacity	Ratio of Specific Heats	Bulk Viscosity
Symbol	*v*	ρ	η	κ_th_	*C*_p_	γ	η_B_
Unit	m/s	kg·m^−3^	Pa·s	W·m^−1^·K^−1^	J·kg^−1^·m^−3^	-	Pa·s
Value	1481	998	0.001	0.599	4184	1.007	0.00286

## Data Availability

Data supporting reported results can be provided by the authors on request.
